# Potential mechanism of ginger *(Zingiber Officinale Roscoe)* in alleviating osteoporosis

**DOI:** 10.3389/fphar.2025.1607278

**Published:** 2025-09-24

**Authors:** Miao Luo, Ning Li, Li Deng, Huan Pei, Funeng Wang, Cong Ma, Pengyi Yang, Kai Yuan, Lvyu Li, Dongdong Qin

**Affiliations:** ^1^ Kunming Municipal Hospital of Traditional Chinese Medicine, Third Affiliated Hospital of Yunnan University of Chinese Medicine, Yunnan University of Chinese Medicine, Kunming, Yunnan, China; ^2^ Key Laboratory of Integrated Chinese and Western Medicine for Chronic Disease Prevention and Control, Yunnan University of Chinese Medicine, Kunming, Yunnan, China; ^3^ Yunnan Yunling Pharmaceutical Resource Innovation Research Institute, Kunming, Yunnan, China; ^4^ Second Clinical Medical College, Yunnan University of Chinese Medicine, Kunming, Yunnan, China; ^5^ Key Laboratory of Traditional Chinese Medicine for Prevention and Treatment of Neuropsychiatric Diseases, Yunnan University of Chinese Medicine, Kunming, Yunnan, China; ^6^ School of Basic Medical Sciences, Yunnan University of Chinese Medicine, Kunming, Yunnan, China

**Keywords:** ginger, osteoporosis, effects, signaling pathways, mechanisms

## Abstract

Osteoporosis (OP), a prevalent metabolic bone disease, significantly compromises patients’ quality of life and overall health. In recent years, with the advancement of natural product research, ginger (*Zingiber Officinale Roscoe*) has garnered attention for its potential anti-osteoporotic effects. This review summarizes the clinical and basic research progress of ginger in alleviating OP, focusing on the mechanisms by which ginger exerts its effects through multiple signaling pathways, including NF-κB, Wnt/β-catenin, MAPK, GSK3β/Nrf2, and RANK/RANKL/OPG. By comprehensively analyzing existing literature, this study explores the research significance and limitations of ginger and its active metabolites in the treatment of OP, and proposes future research directions, aiming to provide theoretical support and reference for the development of novel anti-osteoporotic drugs.

## 1 Introduction

Osteoporosis (OP) is a metabolic disease caused by an imbalance between bone formation and resorption, resulting in decreased bone mass, increased bone fragility, and a higher risk of fractures (Wang et al., 2024). Globally, over 200 million people are affected by OP (Sozen et al., 2017). Epidemiological studies indicate that as populations age, the incidence of OP rises significantly, with projections suggesting that by 2050, more than 50% of brittle fractures in Asia will occur in individuals with OP (Yu and Xia, 2019). While the exact etiology of OP remains unclear, research identifies genetics, endocrine disorders, aging, and intestinal microenvironment disturbances as key contributing factors (Zhang et al., 2024; Wang et al., 2023). These factors are thought to interact through mechanisms involving Reactive oxygen species (ROS), hormonal imbalances, intestinal barrier dysfunction, and other biological pathways (Goldring, 2015; De-Ugarte et al., 2018).

Ginger (*Zingiber Officinale Roscoe*), belonging to the Zingiberaceae family, derived from the root and stem of the ginger plant, holds a prominent place in medicine, nutrition, and culinary traditions (Hu et al., 2023). Globally recognized for its medicinal and therapeutic properties, ginger contains a diverse array of bioactive metabolites, including Gingerols (such as 6-gingerol, 8-gingerol, 10-gingerol), Diarylheptanoids (such as curcumin), Ginger Essential Oil (such as zingiberene, zingerone, shogaols) and others (Zhang et al., 2021; Liu et al., 2019). The metabolite of ginger varies significantly depending on the variety and processing methods. Research indicates that gingerol is the most abundant metabolite in ginger, exhibiting notable antioxidant and anti-inflammatory effects (Sharma et al., 2023). Furthermore, metabolites such as curcumin and gingerone have demonstrated favorable pharmacological properties, including anti-inflammatory and antioxidant activities, in numerous studies (Kaur et al., 2024; Mani et al., 2016).

This review systematically integrates existing clinical and basic research evidence, focusing on the network of five signaling pathways: NF-κB, Wnt/β-catenin, MAPK, GSK3β/Nrf2, and RANK/RANKL/OPG. The dual regulatory mechanisms of ginger bioactive metabolites on OP are elucidated, and the limitations of current research are critically assessed. Future directions and priorities for ginger research are explored, aiming to provide a reference for the development of natural therapies for OP.

## 2 Search strategy

### 2.1 Literature search databases and keywords

A systematic literature search was conducted to identify relevant publications on the potential mechanisms of ginger in alleviating OP. Initially, several key academic databases were selected for the search, including PubMed, Embase, Web of Science and Cochrane. These databases encompass a wide range of medical and biological fields, providing extensive research papers and clinical trial data, which offer valuable information on both basic and clinical research.

During the data retrieval process, specific search term combinations were employed to ensure the acquisition of literature directly related to the association between ginger and OP. Search terms included “osteoporosis,” “signaling pathways,” “clinical,” “ginger,” “Zingiber Officinale Roscoe,” and “gingerol,” among others. These keywords encompass the fundamental properties of ginger and its potential biological mechanisms in the treatment of OP.

### 2.2 Inclusion and exclusion criteria

Clear inclusion and exclusion criteria were established to ensure the quality of the study. The inclusion criteria encompassed clinical or basic research explicitly addressing the effects of ginger or its metabolites on OP. All included studies were required to have a clear experimental design and results, providing scientific evidence for the application of ginger in OP treatment.

Exclusion criteria were equally important. Non-English literature was excluded because it could not provide original data or research findings and might not offer direct information relevant to the review’s topic. Additionally, studies with incomplete data were excluded. Complete data is crucial for drawing reliable conclusions when evaluating the effectiveness of ginger on OP. Strict adherence to the inclusion and exclusion criteria will enhance the scientific rigor and reliability of this review.

### 2.3 Quality control standards

In the quality assessment phase, this study employed a suite of evaluation tools to comprehensively assess the scientific rigor and reliability of various studies. The quality assessment of randomized controlled trials primarily relied on the robustness of the experimental design, including the randomization process, the establishment of control groups, and the implementation of blinding. Furthermore, the rationality of animal study experimental designs was evaluated by observing the differences between experimental and control groups, as well as the selection of experimental animals. For cell experiments, the focus was on the selection of cell lines and the control of experimental conditions. Through comprehensive quality assessment, many confounding factors unsuitable for research were excluded, ensuring the scientific validity and reliability of the conclusions (Table 1).

**TABLE 1:** Ginger and its metabolites for treating osteoporosis.

Metabolites	Drug source	Study type	Subjects	Administration method	Optimal dose	Dose range	Intervention time	Control group	Outcomes	References
6-schogaol	Sigma-Aldrich	*In vitro*	POB and BMC	Co-culture	—	1.25–20 μM	7 days	—	Inhibits prostaglandin E2 synthesis in osteoblasts, reduces interleukin-1-induced RANKL expression, thereby suppressing osteoclast formation	Hwang et al. (2018)
6-schogaol	ChemFace	*In vivo*	Rats	Gavage	20 mg/kg/d	—	14 days	—	Modulates RANKL/OPG balance to mitigate alveolar bone destruction, concurrently enhancing antioxidant enzyme activity and reducing oxidative stress markers	Bezirci et al. (2024)
6-schogaol	Extraction	*In vitro*	MBDM	Co-culture	2.5 μM	0–10 μM	2 days	—	Inhibits RANKL-induced osteoclast differentiation, reduces the formation of multinucleated osteoclasts, and downregulates the expression of key genes (e.g., Nfatc1, c-Fos, Trap, Ctsk)	Kim et al. (2020)
		*In vivo*	Mice	Intraperitoneal injection	10 mg/kg	—	10 days	—	Reduces the expression of IL-1β and TNF-α, effectively mitigating alveolar bone destruction	
Curcumin	Solarbio	*In vitro*	MC3T3-E1 Cells	Co-culture	10 μM	0–100 μM	48 h	—	Inhibits oxidative stress, increases ALP, and promotes osteoblast differentiation	Mohammad et al. (2025)
		*In vivo*	Rats	Intraperitoneal injection	—	10–50 mg/kg	3 months	—	Augments bone mineral density and stimulates trabecular bone formation	
Curcumin	Sigma-Aldrich	*In vitro*	POB	Co-culture	—	0–2 μmol/L	2 h	—	Activates the ERK pathway and inhibits osteoblast apoptosis	Chen et al. (2016)
		*In vivo*	Rats	Gavage	100 mg/kg	—	60 days	—	Enhances bone mineral density, increases b-ALP levels, decreases CTX levels, improves bone biomechanical strength, and repairs trabecular structure	
Zingerone	Sigma	*In vitro*	mMSCs	Co-culture	200 μM	—	7 days	—	Upregulates the expression of miR-590 and downregulates the expression of Smad7 to protect Runx2, thereby promoting osteogenic differentiation	Srinaath et al. (2019)
Zingerone	Alfa Aeasar	*In vitro*	SAOS-2 cells	Co-culture	200 μM	5–200 μM	21 days	—	Promotes the expression of osteogenesis-related genes, such as ALP and Runx2	De Vos et al. (2024)
		*In vitro*	RAW264.7 Cells	Co-culture	200 μM	0.1–200 μM	5 days	—	Inhibits RANKL-mediated osteoclastogenesis	
Zingerone	Sigma-Aldrich	*In vitro*	hBMSCs	Co-culture	200 μM	50–200 μM	72 h	—	Promotes the mRNA and protein expression of osteogenic differentiation markers such as alkaline phosphatase, osteocalcin, osterix, and Runt-related transcription factor 2	Song et al. (2020)
10-gingerol	Extraction	*In vitro*	RAW264.7 Cells	Co-culture	2.5 μM	—	6 days	—	Downregulates osteoclast marker genes (e.g., Oscar, Dc-stamp, Mmp9, and Trap)	Zang et al. (2021)
		*In vivo*	Zebrafish	Direct immersion	1 mg/mL	—	8 days	Prednisolone	Inhibits CTSK enzymatic activity, suppresses osteoclast differentiation	
Cedrol	Sigma Aldrich	*In vitro*	BMMs	Co-culture	20 μm	0–20 μM	5 days	—	Inhibits NF-κB and MAPK signaling pathways, thereby blocking osteoclast gene expression	Xu et al. (2023)
		*In vivo*	Mice	Gavage	20 mg/kg	—	6 weeks	—	Reduces osteoclast numbers and osteoclast-related factors	
Ginger tablets	Dineh	Clinical trial	Patients with osteoporosis	Oral administration	60.88 mg	—	4 months	Curcumin tablets	Reduces ALP levels, increases bone mineral density, and ameliorates inflammation and oxidative stress	Salekzamani et al. (2023)

ALP, alkaline phosphatase; B-ALP, Bone-specific alkaline phosphatase; BMC, bone marrow cells from murine models; BMD, bone mineral density; BMMs, Bone marrow-derived macrophages; CTSK, Cathepsin K; CTX, C-terminal telopeptide of type I collagen; ERK, Extracellular signal-regulated kinase; E2, estrogen; hBMSCs, Human bone marrow stromal/mesenchymal stem cells; MBDM, Murine bone marrow-derived macrophages; mMSCs, Murine mesenchymal stromal cells; OC, osteocalcin; OPG, osteoprotegerin; OSX, Osterix (SP7); POB, primary osteoblasts; RANKL, Receptor activator of nuclear factor kappa B ligand; Runx2, Runt-related transcription factor 2.

## 3 Clinical trials

Salek Zamani’s research team conducted a randomized, triple-blind, placebo-controlled clinical intervention study on postmenopausal women with OP (Salekzamani et al., 2023). The study enrolled 120 patients who were on a baseline treatment of once-weekly alendronate tablets combined with daily calcium and vitamin D3. Subsequently, patients were randomized into three intervention groups (ginger group, curcumin group, and combination group) and a control group (placebo group). The ginger group received two ginger tablets and two placebo tablets daily (each ginger tablet contained approximately 30.44 mg of gingerol). The curcumin group received two curcumin tablets and two placebo tablets daily (each curcumin tablet contained approximately 53.58 mg of curcuminoids). The combination group received two ginger tablets and two curcumin tablets daily, while the control group received four matching placebos that were identical in appearance, smell, and color. After 4 months of intervention, a comprehensive assessment revealed that bone mineral density (BMD) in the femoral neck and lumbar spine increased in the three intervention groups compared to the control group. Furthermore, osteocalcin and alkaline phosphatase levels decreased, serum hs-CRP and IL-6 levels decreased, antioxidant activity was enhanced, and total antioxidant capacity and superoxide dismutase levels increased. While, malondialdehyde levels decreased. These findings suggest that ginger and curcumin can enhance BMD, improve bone turnover markers, reduce inflammation, and enhance antioxidant activity in postmenopausal women with OP.

Clinical trials demonstrate that ginger and its active metabolites, when used as supplements in OP patients, can increase BMD, mitigate inflammatory responses, alleviate ROS damage, and improve overall skeletal health, thereby establishing a robust foundation for long-term bone health. Furthermore, ginger and its active metabolites exhibit anti-inflammatory properties, significantly reducing the expression levels of pro-inflammatory cytokines such as IL-6. By enhancing the body’s total antioxidant capacity and superoxide dismutase activity, while decreasing malondialdehyde levels, ginger effectively mitigates inflammation and ROS-induced damage to bone tissue, thus promoting a healthier microenvironment for bone maintenance and repair.

## 4 Molecular mechanisms

**FIGURE 1:**
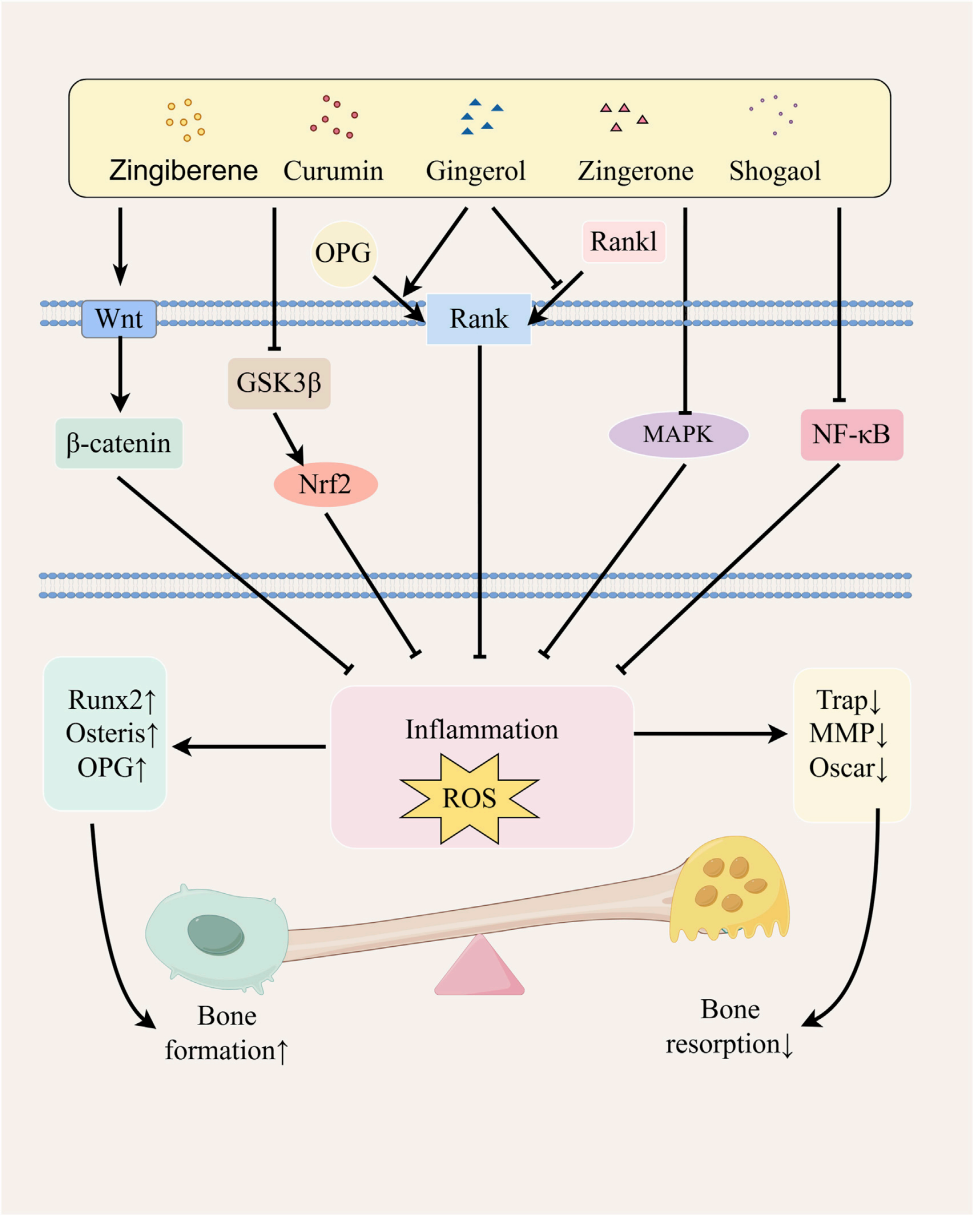
Possible mechanism of ginger in alleviating osteoporosis. Ginger and its active metabolites promote osteoblast differentiation by upregulating the expression of Runx2, Osterix, and OPG genes through the activation of the NF-κB and Wnt/β-catenin signaling pathways, regulation of GSK3β/Nrf2 pathway activity, inhibition of the MAPK pathway, and modulation of the RANK/RANKL/OPG signaling system. Simultaneously, they inhibit osteoclast differentiation by downregulating the expression of TRAP, MMP, and Oscar genes, thereby alleviating osteoporosis. The figure was drawn by Figdraw.

Ginger effectively ameliorates the bone inflammatory microenvironment and oxidative stress levels in OP patients through its anti-inflammatory and antioxidant properties, thereby providing synergistic protection for bone homeostasis (Sharifi-Rad et al., 2017; Mao et al., 2019). Based on these effects, we will subsequently focus on the following five key signaling pathways to systematically elucidate the molecular mechanism underlying the multi-target intervention of ginger in bone metabolic imbalance (Figure 1).

### 4.1 Ginger and its active metabolites inhibit the NF-κB signaling pathway to alleviate OP

The Nuclear Factor Kappa B (NF-κB) signaling pathway is integral to various immune and inflammatory responses (Hoesel and Schmid, 2013). Ginger and its active metabolites regulate inflammation, thereby influencing bone formation processes. Research indicates that the active ginger metabolites, Zingerone inhibits NF-κB signaling in osteoclast precursor cells, reducing bone resorption while suppressing F-actin ring formation and osteoclast activity (Yang et al., 2023). Furthermore, 6-gingerol can repair MG-63 cell damage, suppress IL-6 production, and promote MG-63 cell differentiation into osteoblasts. Additionally, 6-gingerol reduces the inflammatory mediator prostaglandin E2 and hinders osteoclast differentiation associated with inflammation (Fan et al., 2015; Hwang et al., 2018). Zingerone and Curcumin effectively inhibit the differentiation of RAW264.7 cells into osteoclasts by modulating the activation of the NF-κB signaling pathway. Furthermore, these metabolites impede the bone resorption process mediated by ROS (Bharti et al., 2004). Another study confirmed that curcumin suppresses NF-κB and IL-6 expression, blocks the NF-κB signaling pathway, increases BMD, and improves trabecular bone microstructure. These findings highlight curcumin’s ability to modulate the NF-κB pathway and mitigate OP. Moreover, in a rat model of testicular injury induced by cisplatin, ginger juice inhibited the NF-κB signaling pathway, exhibiting potent anti-inflammatory effects (Famurewa et al., 2020). Similarly, 6-Gingerol exerts a protective effect on the intestinal barrier, thereby mitigating the progression of OP, through the modulation of TNF-α induced alterations in tight junction proteins. Specifically, it achieves this by inhibiting the PI3K/Akt and NF-κB signaling pathways, which subsequently influences the upregulation of claudin-2 and the degradation of claudin-1 within the intestinal barrier (Luettig et al., 2016; Wei et al., 2025). Ginger’s suppression of TNF-α further inhibits NF-κB activation, demonstrating anti-inflammatory and anticancer potential (Habib et al., 2008). Furthermore, *in vitro* studies have demonstrated that ginger extract significantly reduces NF-κB activation. Concurrently, the anti-inflammatory effects of ginger constituents have been observed in murine models, closely associated with the inhibition of NF-κB (Xu et al., 2023). These findings provide evidence that ginger exhibits an inhibitory effect on the NF-κB signaling pathway, thereby elucidating the mechanism by which ginger alleviates OP via the NF-ebb signaling pathway.

### 4.2 Ginger and its active metabolites activate Wnt/β catenin signaling pathway to alleviate OP

The Wingless-type MMTV Integration Site Family/β-Catenin (Wnt/β-catenin) signaling pathway represents a critical regulatory mechanism in bone metabolism, and its activation promotes osteogenic differentiation. It promotes the proliferation of pre-osteoblasts, enhancing their differentiation into osteoblasts and increasing bone mass. The Wnt/β-catenin signaling pathway inhibits bone resorption through Wnt signaling ligands, maintaining bone health (Yao et al., 2017). For instance, ginger extract inhibits NF-κB and Wnt pathway activation, protecting against inflammatory arthritis (Oz et al., 2021). It also holds potential in cancer therapy, inducing apoptosis in colorectal cancer cells by suppressing the mTOR and Wnt/β-catenin pathways, this process may also reflect its potential mechanisms in osteocyte regulation. (Wee et al., 2015). In a hormone-induced rat model of OP, curcumin treatment significantly increased femoral BMD, and the expression of Wnt, β-catenin, and osteoprotegerin (OPG) mRNA, while reducing receptor activator of nuclear factor κ-B ligand (RANKL) mRNA expression. These changes restored bone loss and alleviated OP symptoms (Chen et al., 2016). Additionally, curcumin upregulated transcription factors such as Runx2 and OPG, which are critical for osteoblast differentiation, and increased the OPG/RANKL ratio, reactivating the hormone-suppressed Wnt/β-catenin signaling pathway (Cirano et al., 2018; Chen et al., 2016). Similarly, ginger and its bioactive metabolites have been shown to reduce inflammation by modulating the Wnt/β-catenin signaling pathway in various models. Collectively, these findings suggest that ginger and its active metabolites regulate bone metabolism by modulating the Wnt/β-catenin signaling pathway.

### 4.3 Ginger and its active metabolites alleviate OP by affecting the MAPK signaling pathway

The mitogen-activated protein kinase (MAPK) cascade, encompassing serine/threonine protein kinases such as p38MAPK, extracellular signal-regulated kinase (ERK), and c-Jun N-terminal kinase (JNK) subtypes, plays a critical role in bone remodeling processes (Liu et al., 2023; Nagai et al., 2023; Guo et al., 2020). Cedrol (active metabolites of ginger) can effectively alleviate estrogen deficiency-induced OP by inhibiting the differentiation of RANKL-induced bone marrow monocytes into osteoclasts, reducing ROS production, and subsequently blocking the activation of NFATc1, NF-κB, and MAPK signaling pathways (Xu et al., 2023). *In vitro*, gingerol demonstrated a significant inhibitory effect on the expression of MAPK proteins in RAW264.7 cells, consequently suppressing osteoclastogenesis (De Vos et al., 2024). Furthermore, reports have indicated that the primary metabolites of ginger modulate the MAPK signaling pathway across a spectrum of diverse pathologies (Torkzadeh-Mahani et al., 2019). For instance, 6-gingerol alleviates neuropathic inflammation in rats by inhibiting signal transduction from p38 MAPK to NF-κB. It also prevents ROS production and p38 MAPK activation, thereby protecting against intestinal ischemia-reperfusion-induced mucosal damage (Li et al., 2017). Furthermore, 8-gingerol reduces MAPK protein expression, inhibits myocardial cell apoptosis, and improves cardiac injury (Xue et al., 2021). In a mouse model of traumatic brain injury, curcumin suppresses inflammation by downregulating the p38/MAPK pathway (Li et al., 2022). These findings collectively suggest that ginger and its bioactive metabolites may similarly alleviate OP via MAPK pathway modulation.

### 4.4 Ginger and its active metabolites act on the GSK3β/Nrf2 signaling pathway to alleviate OP

The Glycogen Synthase Kinase 3 Beta/Nuclear Factor Erythroid 2–Related Factor 2 (GSK3β/Nrf2) signaling pathway is fundamentally implicated in cellular signaling cascades and the modulation of ROS (Qi et al., 2015; Rana et al., 2012). These findings suggest that ginger may not only act directly on the skeletal system during OP treatment but also support bone health through its antioxidant properties, reduction of ROS, and inhibition of inflammatory factor production (Zammel et al., 2022). Research by Li et al. (2020) demonstrated that curcumin inhibits GSK3β and activates Nrf2, thereby suppressing ROS production. This cascade effectively mitigates ROS, consequently establishing a robust protective mechanism for osteoblasts. Similarly, multiple studies have shown that ginger exerts therapeutic effects via the Nrf2 pathway. For example, 6-gingerol activates Nrf2, enhancing antioxidant capacity and potentially preventing Alzheimer’s disease 6-Shogaol alleviates allergic dermatitis by activating the Nrf2 signaling pathway, alleviating ROS, and inhibiting immune mediators (Park et al., 2016), while ginger oleoresin induces Nrf2 nuclear translocation, decreases ROS generation, and protects mesenchymal stem cells from ionizing damage (Ji et al., 2017). These findings suggest that the therapeutic efficacy of ginger and its bioactive metabolites in the context of OP may be mediated by the suppression of the GSK3β signaling pathway and the concurrent upregulation of the Nrf2 signaling pathway.

### 4.5 Ginger and its active metabolites regulate the RANK/RANKL/OPG signaling pathway to alleviate OP

The Receptor Activator of NF-κB/RANK Ligand/Osteoprotegerin (RANK/RANKL/OPG) signaling pathway plays a pivotal role in bone remodeling. RANKL and OPG are transmembrane proteins, while RANK functions as a receptor expressed on osteoclasts. OPG competes with RANKL for binding to RANK, thereby inhibiting osteoclast activation and differentiation as well as suppressing the bone-resorbing activity of mature osteoclasts (Yasuda, 2021). The Bezirci research group have observed that 6-shogaol significantly reduces RANKL levels and MDA content (Bezirci et al., 2024). It mitigates bone loss by modulating the RANKL/OPG ratio and exerting antioxidant effects. Furthermore, the levels of RANKL were significantly reduced, and MDA content was decreased. The team concluded that 6-shogaol mitigates bone loss by modulating the RANKL/OPG ratio and exerting antioxidant effects. Additionally, in fractured rat models, 6 weeks of curcumin treatment reduced RANK and RANKL expression in the femur, decreased the RANKL/OPG ratio, and inhibited osteoclastogenesis, leading to enhanced bone formation over resorption (Ilka et al., 2022). These findings suggest that the beneficial effects of ginger on OP may be attributed to its ability to regulate the RANK/RANKL/OPG signaling pathway through its bioactive metabolites.

## 5 Discussion

In recent years, ginger has garnered increasing attention for its role as a traditional medicinal plant in the treatment of OP. Through literature review and data synthesis, we demonstrate that ginger and its active components may alleviate bone metabolic imbalances by intervening in the following five key signaling pathways. These five signaling pathways include NF-κB, Wnt/β-catenin, MAPK, GSK3β/Nrf2, and RANK/RANKL/OPG. The NF-κB pathway plays a role in inflammation and bone resorption. Ginger metabolites, such as cedrol, can reduce bone resorption by inhibiting NF-κB activation (Xu et al., 2023) The Wnt/β-catenin pathway plays a key role in bone formation, and the active metabolites in ginger can promote the differentiation and function of osteoblasts by regulating this pathway (Jiang et al., 2021). In addition, the MAPK pathway is closely related to cell proliferation and differentiation, and ginger metabolites can affect the biological behavior of bone cells by regulating this pathway (Yang et al., 2023). The GSK3β/Nrf2 pathway is closely related to the regulation of oxidative stress. Studies have shown that ginger metabolites can enhance antioxidant capacity by activating Nrf2, thereby protecting bone tissue from oxidative damage (Li et al., 2020). Finally, the RANK/RANKL/OPG signaling pathway is a key regulatory mechanism for bone remodeling. Ginger metabolites can inhibit the RANKL/OPG balance and antioxidant status, reduce bone resorption rate, and thus alleviate bone loss (Ilka et al., 2022). Through a comprehensive analysis of these signaling pathways, the multi-target effects of ginger and its active metabolites in the treatment of OP are emphasized.

### 5.1 Research significance

Studying the potential mechanism of ginger in alleviating OP has important clinical and basic research significance. With the acceleration of global aging, OP has become a common public health problem, especially in postmenopausal women (Nicholson et al., 2025). Although traditional drug treatment is effective, it is often accompanied by side effects such as nausea and emotional abnormalities, which brings many challenges to the treatment of patients (Kellar et al., 2020). As a traditional medicine plant, ginger has complex metabolites and involves a variety of bioactive metabolites. It regulates the balance of bone metabolism through multiple targets and is a safe and effective natural medicine (Kiyama, 2020).

### 5.2 Research limitations

This review is subject to certain limitations. Firstly, there is potential for bias in the literature coverage. To ensure retrieval efficiency, this review primarily relied on English databases (such as PubMed, Embase, Web of Science and Cochrane) for systematic searches, which may have omitted research literature not included in mainstream databases. Future research should be expanded to other multilingual databases, such as CNKI and ClinicalTrials.gov. Secondly, the depth of discussion on signaling pathway mechanisms needs to be improved. This review only provides a relatively brief analysis of some complex molecular mechanisms, not involving the interactive regulation of multiple keys signaling pathways. In the future, we should conduct special research on these core pathways, combining bioinformatics analysis and experimental verifications to provide more targeted mechanistic evidence for clinical decision-making.

In evaluating studies on the impact of ginger on OP, we also identified several limitations in experimental designs. Firstly, the sample sizes were small, and the observation periods were short. Some clinical trials, despite being designed as triple-blind randomized controlled trials, included only 120 subjects with an observation period of just 4 months (Salekzamani et al., 2023). This may not fully reflect the true effects of ginger on bone mineral density and related biomarkers, thereby affecting the reproducibility and statistical significance of the results. Secondly, the elucidation of specific mechanisms is still insufficient. Although the bioactive metabolite of ginger, gingerol, has demonstrated potential in inhibiting osteoclast formation and promoting osteoblast formation, the observational data is insufficient. Furthermore, the exploration of specific mechanisms remains inadequate, lacking support from large-scale, reproducible experiments (Fan et al., 2015; Hwang et al., 2018).

### 5.3 Future research direction

In the investigation of ginger’s advancements in OP research, future research directions should concentrate on two key areas to further elucidate its mechanisms and clinical application potential. Firstly, it is imperative to expand the sample size and extend the duration of studies. Current clinical studies often involve small sample sizes and short observation periods, which limits a comprehensive evaluation of ginger extract’s efficacy. Although existing research indicates ginger’s potential in improving physiological indicators associated with OP (Salekzamani et al., 2023), the small sample sizes make it difficult to draw universally applicable conclusions, and the short observation periods hinder the assessment of ginger’s long-term effects and potential side effects. Therefore, future research necessitates larger sample sizes and extended observation periods to obtain more accurate data. Secondly, it is necessary to deepen the study of molecular mechanisms. Although existing research has confirmed that ginger affects bone metabolism by inhibiting osteoclast activity and promoting osteoblast differentiation (Hwang et al., 2018; Kim et al., 2022; Gupta et al., 2025; Fan et al., 2015), the mechanisms by which its metabolites regulate the network of bone metabolism at the molecular level have not been elucidated, and this critical mechanism is systematically lacking, severely limiting the potential for drug development. There is an urgent need to deeply explore the role of ginger’s active metabolites in signaling pathways and identify gene targets.

As the core active metabolite of ginger, gingerol exhibits significant antioxidant and anti-inflammatory properties as well as multi-target signaling pathway regulatory capabilities (Xu et al., 2023). More importantly, it intervenes in the pathological process of OP by bidirectionally regulating bone metabolism balance, i.e., inhibiting osteoclast differentiation while simultaneously activating osteoblast differentiation (Zang et al., 2021; Fan et al., 2015). Based on its multi-pathway synergistic mechanism and the safety advantages, it is recommended to prioritize gingerol as a key breakthrough drug for the prevention and treatment of OP, and to conduct in-depth analysis of mechanisms underlying its regulation of bone metabolism network.

## 6 Conclusion

Ginger significantly alleviates the pathological process of OP by coordinately modulating five major signaling pathways, including NF-κB, Wnt/β-catenin, MAPK, GSK3β/Nrf2, and RANK/RANKL/OPG. This multi-target synergistic mechanism offers a unique advantage for developing safe and effective natural anti-osteoporosis drugs, particularly suitable for elderly and postmenopausal women who are intolerant to traditional drugs. In spite of the promising prospects of current research, deficiencies in experimental design and mechanistic exploration still need to be addressed. Through further high-quality research and standardized extraction processes, we anticipate better application of ginger and its active metabolites in clinical practice, promoting its development in the treatment of OP. Simultaneously, this provides a new perspective and approach for the modernization of traditional Chinese medicine, promoting the integration of traditional drugs with modern medicine to provide patients with more comprehensive treatment options.
